# Exploring genetic resistance to infectious salmon anaemia virus in Atlantic salmon by genome-wide association and RNA sequencing

**DOI:** 10.1186/s12864-021-07671-6

**Published:** 2021-05-13

**Authors:** O. Gervais, A. Barria, A. Papadopoulou, R. L. Gratacap, B. Hillestad, A. E. Tinch, S. A. M. Martin, D. Robledo, R. D. Houston

**Affiliations:** 1 The Roslin Institute and Royal (Dick) School of Veterinary Studies, University of Edinburgh, Edinburgh, UK; 2 Benchmark Genetics Norway, Sandviksboder 3A, N-5035 Bergen, AS Norway; 3 Benchmark Genetics Ltd, Benchmark House, 8 Smithy Wood Drive, Sheffield, S35 1QN UK; 4 School of Biological Sciences, University of Aberdeen, Aberdeen, UK

**Keywords:** Disease resistance, RNA-Seq, Fish, Aquaculture, *Salmo salar*, TRIM25, GWAS, Heritability

## Abstract

**Background:**

Infectious Salmonid Anaemia Virus (ISAV) causes a notifiable disease that poses a large threat for Atlantic salmon (*Salmo salar*) aquaculture worldwide. There is no fully effective treatment or vaccine, and therefore selective breeding to increase resistance to ISAV is a promising avenue for disease prevention. Genomic selection and potentially genome editing can be applied to enhance host resistance, and these approaches benefit from improved knowledge of the genetic and functional basis of the target trait. The aim of this study was to characterise the genetic architecture of resistance to ISAV in a commercial Atlantic salmon population and study its underlying functional genomic basis using RNA Sequencing.

**Results:**

A total of 2833 Atlantic salmon parr belonging to 194 families were exposed to ISAV in a cohabitation challenge in which cumulative mortality reached 63% over 55 days. A total of 1353 animals were genotyped using a 55 K SNP array, and the estimate of heritability for the trait of binary survival was 0.13–0.33 (pedigree-genomic). A genome-wide association analysis confirmed that resistance to ISAV was a polygenic trait, albeit a genomic region in chromosome Ssa13 was significantly associated with resistance and explained 3% of the genetic variance. RNA sequencing of the heart of 16 infected (7 and 14 days post infection) and 8 control fish highlighted 4927 and 2437 differentially expressed genes at 7 and 14 days post infection respectively. The complement and coagulation pathway was down-regulated in infected fish, while several metabolic pathways were up-regulated. The interferon pathway showed little evidence of up-regulation at 7 days post infection but was mildly activated at 14 days, suggesting a potential crosstalk between host and virus. Comparison of the transcriptomic response of fish with high and low breeding values for resistance highlighted TRIM25 as being up-regulated in resistant fish.

**Conclusions:**

ISAV resistance shows moderate heritability with a polygenic architecture, but a significant QTL was detected on chromosome 13. A mild up-regulation of the interferon pathway characterises the response to the virus in heart samples from this population of Atlantic salmon, and candidate genes showing differential expression between samples with high and low breeding values for resistance were identified.

**Supplementary Information:**

The online version contains supplementary material available at 10.1186/s12864-021-07671-6.

## Background

The demand for high-quality animal protein for human diets has increased steadily during the last decades and is expected to accelerate over the next thirty years, in parallel to human population growth [1]. When paired with the challenges of climate change and increased competition for land use [2], a sustainable increase in farmed animal protein production efficiency is required to meet the global food security challenge [3, 4]. Aquaculture is typically resource-efficient, with high rates of feed efficiency and protein retention compared to terrestrial livestock [5], and is expected to play a major role feeding the world in the coming years. While aquaculture production has risen steadily in the recent decades [6], it can also be high-risk, in part due to infectious diseases, which pose major threats to entire production systems, with downstream impacts on efficiency and sustainability.

One such disease threat for farmed Atlantic salmon (*Salmo salar*) is infectious salmon anaemia (ISA), caused by an aquatic orthomyxovirus of the same name (ISAV). ISAV is an enveloped negative-sense single stranded RNA virus member of the family *Orthomyxoviridae*, and therefore closely related to influenza viruses. Viruses of this family share similar strategies of infection, using haemagglutinin activity to enter the cells and fusion activity to escape the lysosome, followed by viral RNA replication in the nucleus of the host cell and modulation of host immune responses [7–9]. The genome of ISAV is divided in 8 segments that encode at least 10 different proteins, and the virus can be divided in two groups, the low virulence ISAV-HPR0 and the virulent ISAV-HPRΔ, which has a deletion in the highly polymorphic region of the haemagglutinin-esterase gene [10]. ISA is classified as a list II disease by the EU fish health directive and as a notifiable disease by the World Organisation for Animal Health [11], which means that entire stocks have to be culled upon detection of the virus to avoid the spread to nearby farms. While outbreaks were first detected in Norway, ISA has been observed in all major salmon producing countries [12–18]. Just 2 years after its first detection in Chile in 2007, ISA caused the collapse of the salmon aquaculture industry of the country, reducing Atlantic salmon production by ~ 75% in two consecutive years [19]. The most characteristic clinical sign of the disease is severe anaemia, often accompanied by lack of appetite and lethargic behaviour [19]. In production settings, a severe ISA outbreak can cause mortalities of above 90% [20]. Currently there are no effective treatments against ISAV, and available vaccines are typically only partially protective [21].

The use of genetic and genomic technologies is becoming an integral part of efforts to reduce the frequency and severity of disease outbreaks in aquaculture species [22]. Genomic selection exploits both between and within family genetic variation to improve the innate resistance of aquaculture stocks via selective breeding, with cumulative benefits every generation [21]. Several studies have shown that host resistance to ISAV has a significant additive genetic component in Atlantic salmon, with heritability estimates ranging from 0.13 to 0.40 [23–28]. Furthermore, studies using molecular markers to investigate the genetic architecture underlying this heritability have revealed putative minor QTL [28–30], and a comparative genomic analysis highlighted potential underlying genes [31]. Several studies have also examined the host response to ISAV by profiling gene expression in tissues and cell lines [32–37]. Generally, these studies have reported a notable up-regulation of innate immunity which did not confer complete protection from the impact of the virus, and which was less marked in vaccinated or secondary-infected fish. Notably, salmon immune responses to ISAV have been reported to be tissue-dependant and tightly regulated by viral transcription [37].

Genetic improvement by selective breeding is limited by the existing additive genetic variation for the trait of interest in the population, and the ability to efficiently measure the trait, which limits the accuracy of selection and therefore genetic gain. The detection of functional genes and variants controlling disease resistance, as well as a better understanding of the genomic mechanisms underpinning disease resistance, can contribute to improve the efficiency of aquaculture breeding programmes by improvement of genomic selection methods [22]. Furthermore, this information can feed into genome editing efforts to enhance disease resistance, whether it is exploiting existing genetic variation or generating de novo mutations based on the functional basis of disease resistance [38, 39]. One route to achieving this is to integrate transcriptomic data with genetic mapping data to identifying putative functional genes and pathways connected to resistance, and this approach has been applied for genetic resistance to viral and parasitic diseases in Atlantic salmon [40, 41].

To assess the potential for selection of ISAV resistance in a commercial Atlantic salmon population and gain insight into the functional genetic basis of the trait, a large scale ISAV disease challenge in 2833 Atlantic salmon parr belonging to 194 families of the SalmoBreed and StofnFiskur strains was performed. A total of 1353 fish were genotyped for 55 K SNP markers, and RNA sequencing was performed on subsets of the challenged population with divergent breeding values for resistance. These datasets were then used to: i) evaluate the heritability of resistance to ISAV in a commercial Atlantic salmon population, ii) assess the genetic architecture of the trait using a genome-wide association study (GWAS), and iii) compare the transcriptomic responses to infection and if this response varied between resistant and susceptible animals.

## Results

### Disease challenge and genetic parameters of ISAV resistance

**Fig. 1:**
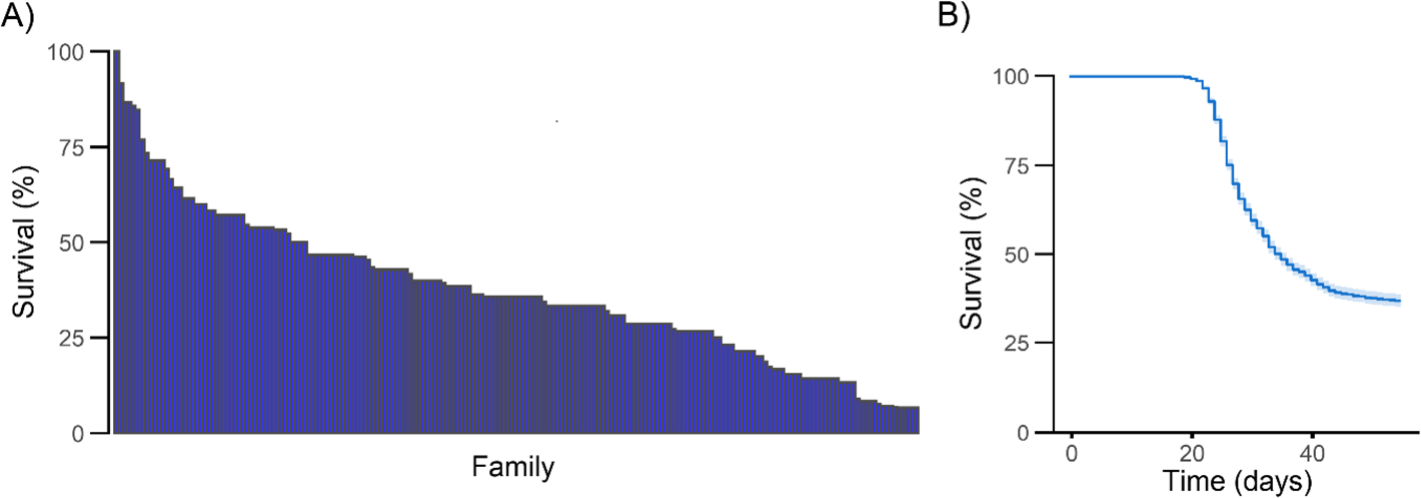
Patterns of mortality observed during the ISAV challenge. **a** Percentage of survival for each full-sibling family at the end of the challenge, and **b**) percentage of surviving fish in the population throughout the duration of the challenge

The ISAV cohabitation challenge on 2833 fish belonging to 194 families (15.9 ± 4.5 fish per family) from Benchmark Genetics commercial breeding programme showed substantial variation in mortality rate between families (Fig. 1a), with values ranging from 7 to 100%, suggesting the presence of a genetic component underlying resistance to ISAV in this population. Mortalities began at day 19 and reached 63%, with most mortalities occurring between day 22 and 28 (Fig. 1b). The pedigree-based heritability for resistance to ISAV was estimated to be 0.13 ± 0.05.

### Genetic architecture of ISAV resistance

**Fig. 2:**
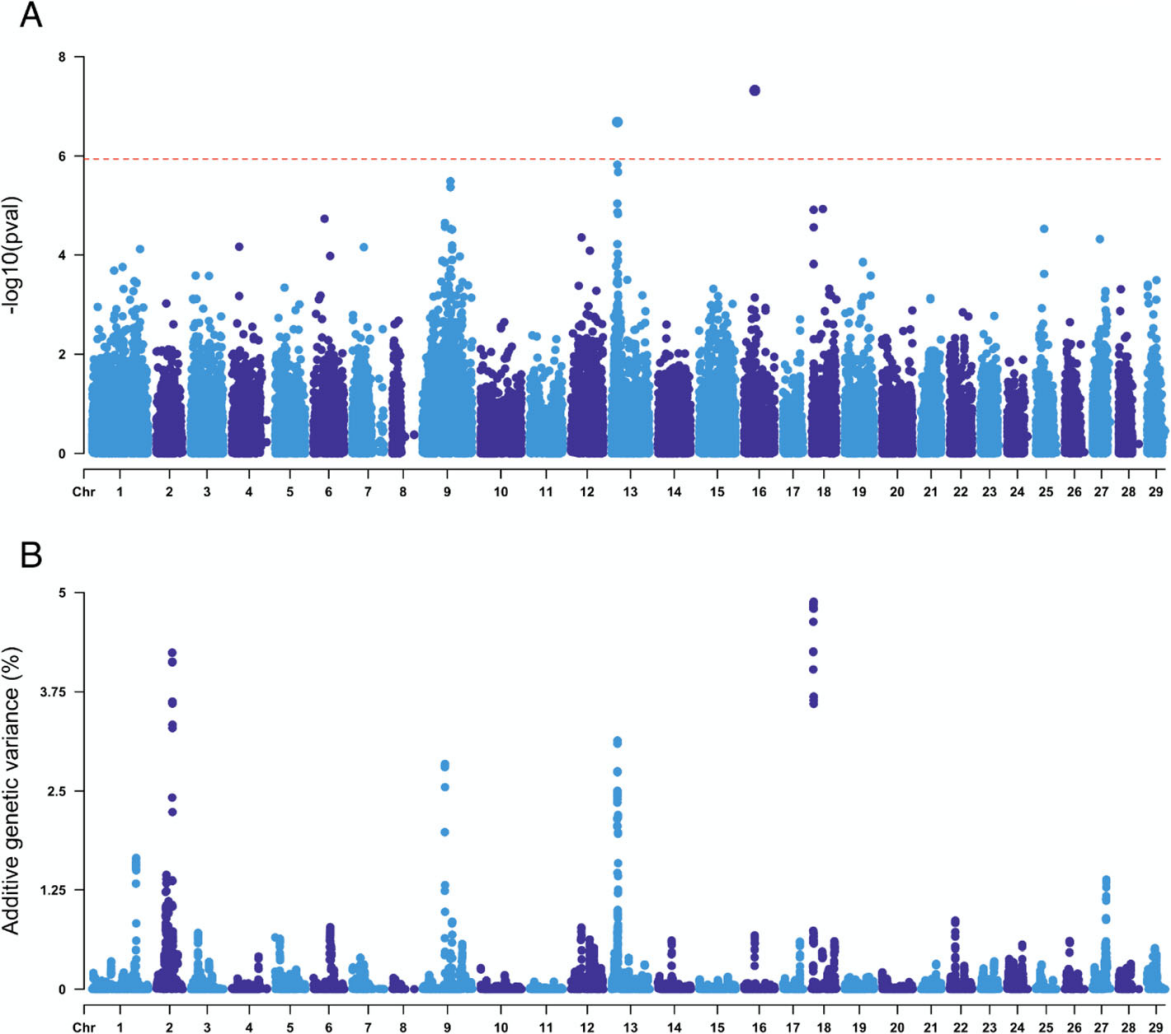
Weighted single-step genome-wide association analyses for resistance to ISAV in the challenged Atlantic salmon population. **a** Shows the *p*-value for each SNP in a single SNP GWAS, and the red dotted horizontal line represents the significance threshold (*p*-value < 0.05 after Bonferroni correction); **b** shows the percentage of additive variation explained by windows of 20 consecutive SNPs. 17 SNPs are placed in scaffolds not assigned to chromosomes (ICSASG_v2; Lien et al. 2016) and are not shown. These unassigned SNPs explained less than 0.01% of the genetic variance and were not significantly associated with resistance to ISA

A subset of the challenged population (*n* = 1353; 194 families; 7.0 ± 3.4 fish per family) was genotyped using a 55 K SNP array. After QC processing, a total of 43,346 SNPs and 1103 fish remained for downstream analyses. Genomic heritability estimated using the weighted single-step GBLUP model was 0.33 ± 0.04, which is notably higher than the pedigree estimate. The single SNP genome-wide association analysis revealed a significant QTL in chromosome Ssa13 (Fig. 2a, Table 1, Supplementary Table 1; a single SNP in chromosome Ssa16 also reached the significance threshold, but was not supported by other SNPs in the region and explained a very small percentage of the total genetic variance, therefore it was not considered). The significant QTL in chromosome Ssa13 explained ~ 3% of the genetic variance in resistance to ISAV, while five other genomic regions each explained more than 1%, with the largest-effect detected in Ssa18 (4.8%) (Fig. 2b, Table 1, Supplementary Table 1). Overall, the data supported a polygenic basis for host resistance to ISAV, with minor effect loci distributed across several chromosomes.

**Table 1:** Top 10 SNPs associated with resistance to ISAV according to *p*-value and percentage of genetic variation explained (*p*-value < 0.05)

Chr.	Position	Pval	Gen.Var. (%)	Chr.	Position	Pval	Gen.Var. (%)
16	31,177,052	4.77E-08	0.01	18	5,920,835	1.52E-04	4.80
13	16,490,837	2.06E-07	3.13	2	45,107,000	9.75E-03	4.13
13	16,491,495	1.50E-06	1.47	18	5,928,874	1.23E-05	3.60
13	16,449,439	9.14E-06	2.75	13	16,490,837	2.06E-07	3.13
18	5,928,874	1.23E-05	3.60	13	16,474,523	1.08E-03	3.09
13	18,222,026	1.49E-05	1.23	9	63,708,663	5.62E-04	2.84
13	18,189,947	1.28E-04	1.97	9	63,494,740	3.04E-02	2.82
18	5,920,835	1.52E-04	4.80	9	63,493,152	9.60E-03	2.82
13	18,220,651	2.34E-04	1.25	9	63,275,439	8.06E-03	2.80
9	63,755,526	4.62E-04	2.55	13	164,49,439	9.14E-06	2.75

### Transcriptomic response to ISAV

**Fig. 3:**
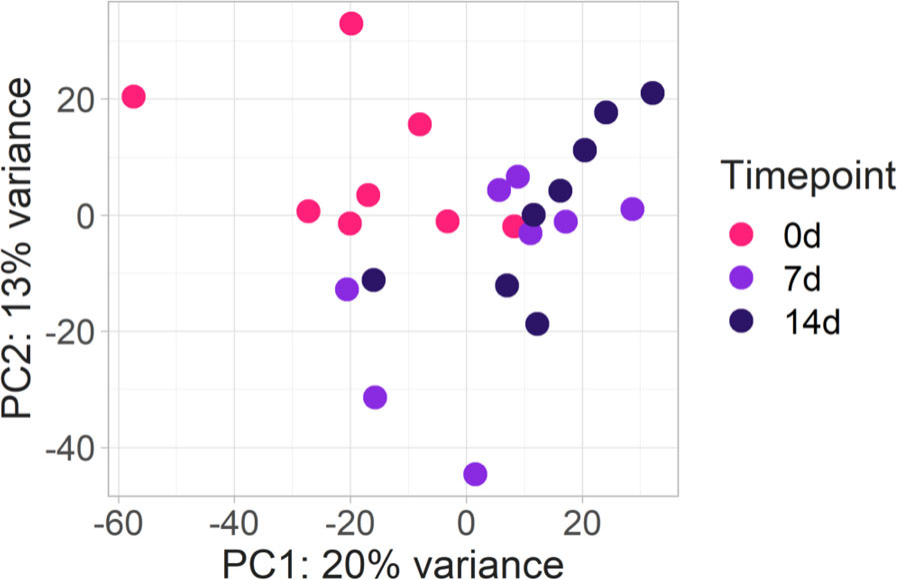
Principal Components Analysis showing the clustering of the heart RNA-Seq data

Based on the genomic estimated breeding values for resistance to ISAV and family mortalities, 4 resistant and 4 susceptible animals were selected at each of three timepoints (0, 7 and 14 days post infection (dpi)). These early timepoints were selected to increase the chance of detecting potential genetic resistance mechanisms. The average GEBVs of resistance to ISAV for the more resistant and more susceptible groups across all timepoints were 0.05 and 0.41 (survival = 0, mortality = 1), respectively, with average family survival rates of 64 and 17% for each group. The transcriptome of the heart samples from these animals was sequenced using Illumina technology, obtaining an average of 51 million of reads per sample. Principal component analyses highlighted that control and infected samples clustered separately according to the two first principal components, which explained 20 and 13% of the total variance (Fig. 3). However, there was no clear differentiation between the challenged timepoints, nor between the resistant and susceptible samples (Supplementary Fig. 1).

**Fig. 4:**
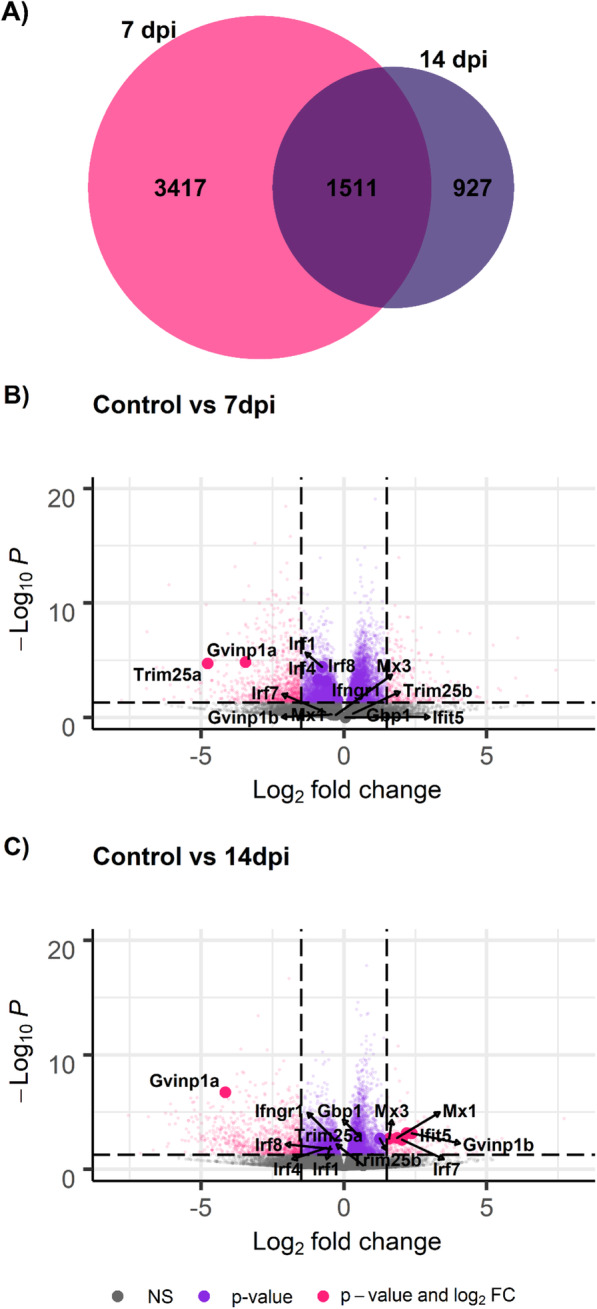
Differential expression of transcripts between ISAV-infected and control fish. **a** Venn diagram depicting the number of common and unique genes showing differential expression at 7 and 14 days compared to control. **b** Volcano plot showing the differential expression and differentially expressed interferon genes in control vs 7 dpi, and **c** controls vs 14 dpi. Each point in the plots represents a gene, with its log_2_ fold change in the x-axis and its –log_10_ *p*-value in the y-axis. Genes are classified in 4 categories depending on their FC and FDR corrected *p*-value: i) grey = *p*-value > 0.05; ii) purple = *p*-value < 0.05 and log_2_ fold change < |1.5|; iii) pink = *p*-value < 0.05 and log_2_ fold change > |1.5|

Although the differentiation between the sampling timepoints based on all transcript data was not clear cut, there was a notable response to ISAV observed in the heart samples both at 7 and 14 dpi, with 4927 and 2437 differentially expressed genes when compared to controls, respectively (Fig. 4 & Supplementary Table 2). A large proportion of the genes differentially expressed at 7 days were also differentially expressed at 14 dpi (1511 genes; Fig. 4a).

Several genes in the interferon pathway are mildly down-regulated at 7 dpi (Fig. 4b), suggesting an initial repression of the antiviral pathway early in infection, as shown by several interferon regulatory factor (IRF) mRNAs being significantly lower in infected samples compared to controls (albeit with small fold change). However, at 14 dpi, several interferon response genes showed up-regulation, such as Mx1, Mx3 or one copy of Interferon-induced Very Large GTPase 1 (GVINP1, another copy of the gene is down-regulated) (Fig. 4c). Other well-characterised immune genes showed differential expression, such as Tumor Necrosis Factor alpha, were down-regulated at 7 days but not at 14 dpi. Down-regulation of numerous complement genes was observed at both timepoints, and in fact KEGG pathway enrichment analyses (Table 2 & Supplementary Table 3) revealed a clear and increasing down-regulation of the complement and coagulation cascades pathway during infection. Almost all the complement and coagulation cascade genes showing putative downregulation at 7 dpi presented even larger negative fold changes in expression at 14 dpi, and additional genes from the same pathway showed statistically significant down-regulation (Supplementary Table 4). Similarly, a consistent up-regulation of numerous metabolic processes is observed at both 7 and 14 dpi. Several of the pathways typically activated during innate immune response to viruses, such as interferon, interleukin or inflammation pathways, are not enriched amongst the set of up- or down-regulated genes, although the pathway HTLV-I (human T-lymphotropic virus type 1) infection is down-regulated at 7 dpi, and so are certain signalling pathways closely related to innate immune responses such as FoxO and mTOR signalling.

**Table 2:** Selected KEGG pathways identified as enriched among differentially expressed genes

**7 dpi**							
**Up-regulated**			**Down-regulated**				
**KEGG**	**N**	**FE**	**p**	**KEGG**	**N**	**FE**	**p**
Carbon metabolism	83	6.01	10^−16^	Complement and coagulation cascades	23	2.73	0.002
Aminoacyl-tRNA biosynthesis	39	2.98	10^−13^	FoxO signalling pathway	36	1.90	0.010
Citrate cycle (TCA cycle)	38	2.27	10^−11^	HTLV-I infection	50	1.67	0.012
**14 dpi**							
**Up-regulated**			**Down-regulated**				
**KEGG**	**N**	**FE**	**p**	**KEGG**	**N**	**FE**	**p**
Carbon metabolism	75	9.31	10^−30^	Complement and coagulation cascades	57	12.76	10^− 36^
Glycolysis / gluconeogenesis	31	4.50	10^−11^	*Staphylococcus aureus* infection	24	5.31	10^−15^
Biosynthesis of amino acids	35	6.99	10^−10^	Systemic lupus erythematosus	17	7.17	10^−5^

*KEGG* KEGG pathway, *N* Number of genes differentially expressed assigned to the corresponding KEGG pathway, *FE* Fold enrichment, *p* False discovery rate corrected *p*-value.

### Genomic signatures of resistance to ISAV

**Fig. 5:**
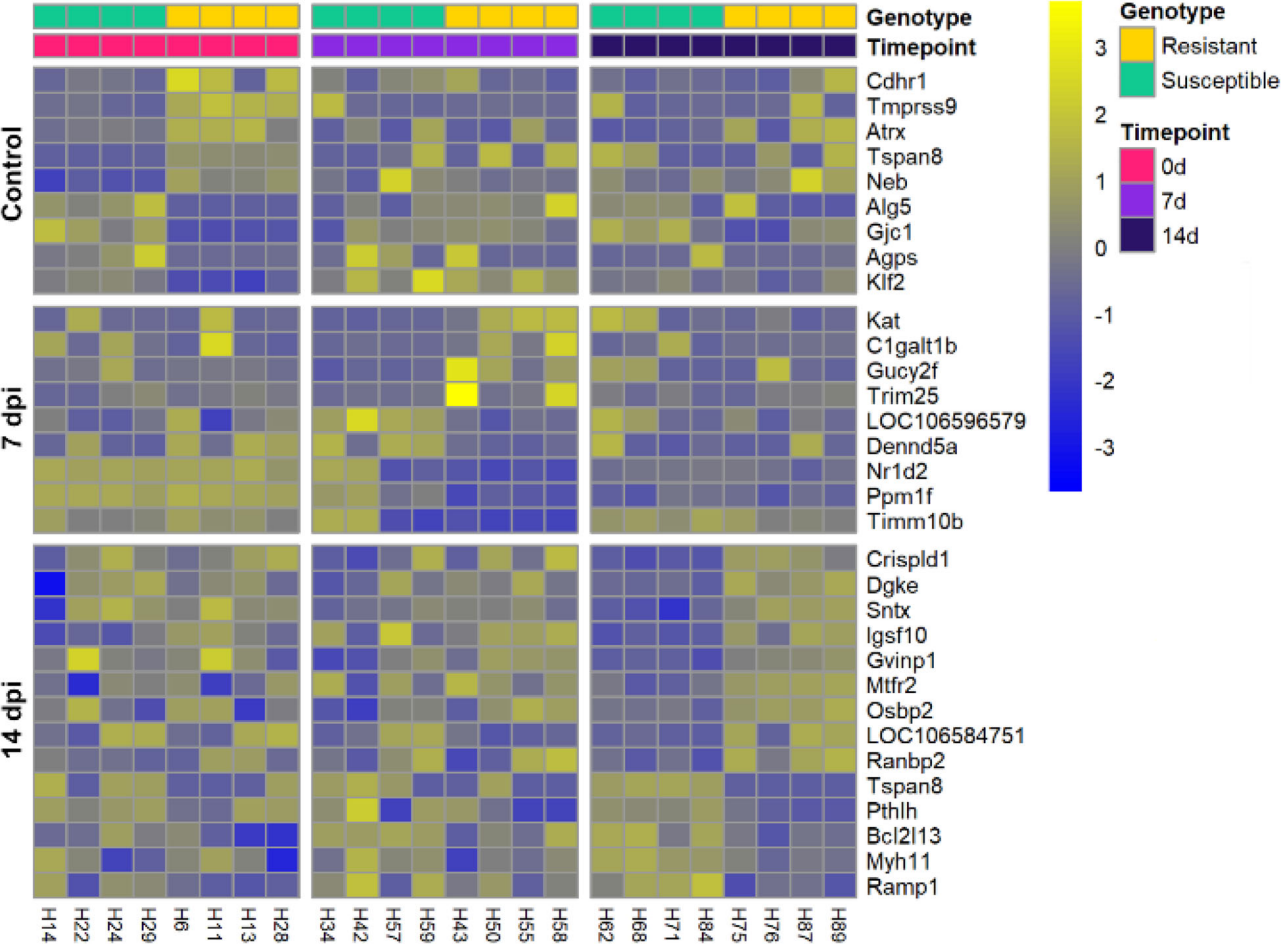
Heatmap showing the patterns of expression of genes differentially expressed between resistant and susceptible fish in each sample at all of the three timepoints

To assess the functional genomic basis of resistance, 4 fish of high resistance breeding values and 4 fish of low resistance breeding values were compared at each of the three timepoints (pre-challenge, 7 and 14 dpi). There were a relatively small number of significantly differentially expressed genes between resistant and susceptible fish (13–18 DEG per timepoint; Fig. 5 & Supplementary file 5). However, these included innate immune response genes of interest such as E3 ubiquitin/ISG15 ligase TRIM25 (involved in innate immune defence against viruses; more expressed in resistant fish at 7 dpi, logFC = 3.90, albeit mainly showing up-regulation in two resistant fish), interferon-induced very large GTPase 1 (more expressed in resistant fish at 14 dpi, logFC = 1.31), or transcription factor Kruppel-like factor 2 (regulates inflammatory processes; less expressed in resistant controls, logFC = − 1.03) (Fig. 5).

### Integration of genetic association and gene expression

**Fig. 6:**
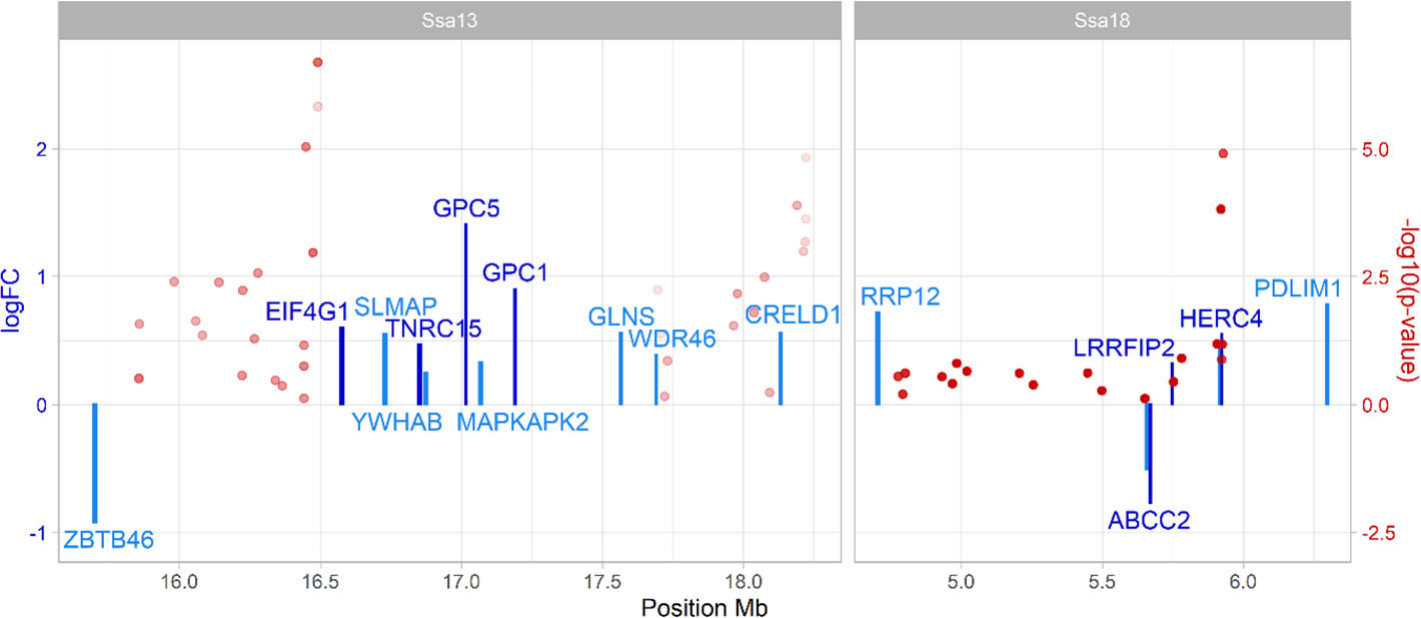
Genetic association and differential expression results in the genomic region with the lowest *p*-value (Ssa13) and the one explaining the highest percentage of genetic variation (Ssa18). The SNPs explaining at least 1% of the genetic variance in each region are shown as red dots, with the shading representing the percentage of genetic variance explained (darker points explaining more variance); the SNPs are placed on the y-axis according to their GWAS –log 10 p-values (association with resistance to ISAV). The log2 fold change of the genes showing differential expression versus controls at 7 dpi (light blue) or 14 dpi (dark blue) are shown as bars, with the scale on the left y-axis

To inform potential genes and mechanisms underlying putative ISAV resistance QTLs, the gene expression results were overlaid onto the main genome-wide significant QTL (Ssa13), and the genomic region explaining the most genetic variance (Ssa18) (Fig. 6). For Ssa13, the Eukaryotic translation initiation factor 4 gamma 1 (EIF4G1), up-regulated 14 days post infection, is one of the closest genes to the most significant SNPs. For Ssa18, the most significant SNPs overlap with the probable E3 ubiquitin-protein ligase HERC4, which is up-regulated in response to infection at both 7 and 14 dpi. None of the genes showing differential expression between resistant and susceptible animals co-located with the putative QTL.

## Discussion

In this study, the genetic and genomic basis of resistance to ISAV in Atlantic salmon was characterised in a large population of Atlantic salmon parr derived from 194 families of a commercial breeding programme. The trait of binary survival (reflecting host resistance) shows a moderate genetic component and is therefore amenable to selection; heritability of resistance was estimated at 0.13 and 0.33 with the pedigree and genomic relationship matrices, respectively. Higher heritability estimates using genomic relationships have been observed compared to pedigree-based estimates in previous studies investigating disease resistance in aquaculture species [42, 43], potentially due to high linkage disequilibrium due to recent selective breeding causing overestimation of additive genetic variance using genomic markers [43]. Nonetheless, these results are in line with the range of previous heritability estimates for resistance to ISAV in Atlantic salmon (0.13–0.40 [23–28]). The genetic architecture of resistance to ISAV in this population was polygenic, and major QTL were not observed, as is common for disease resistance traits [43–48]. However, one genome-wide significant QTL was detected on Ssa13, and an additional three genomic regions explaining > 2.5% of the genetic variation in resistance to ISAV were detected (Ssa18, Ssa02, Ssa09).

A previous study investigating host resistance to ISAV also detected a SNP marker associated with survival to ISAV on Ssa13, however the most signifiant SNPs in the two putative QTL are almost 50 Mb apart [28]. Together with two additional previous studies, putative QTL affecting resistance to ISAV have been mapped on 12 different chromosomes [28–30], none of which were significantly associated with resistance in the current study. While the highly polygenic nature of ISAV and the different origins of the populations studied can explain the different heritabilities and lack of overlap between GWAS studies, it is also plausible that differences in the challenge model have a large effect over the trait of resistance to ISAV. One of the studies employed a challenge model based on intraperitoneal injection [30], which ensures that all fish are infected at the same time but neglects mucosal barriers that may play an important role in resistance. Furthermore, previous studies using co-habitation have used a higher proportion of ‘Trojan’ fish, which may result in a higher infection pressure and differences in host response [37]. Finally, the genetic correlation between resistance to ISAV in freshwater (this study, [28]) and seawater [29] should be addressed in future studies; the life stage of the fish can have an important impact on resistance to ISAV and could also explain some of the differences between studies.

The only genomic region with a significant association with resistance to ISAV in our study was found in chromosome Ssa13 (~ 16,490,837 bp), explaining ~ 3% of the genetic variance. The closest gene to the most significant SNPs showing differential expression is the Eukaryotic translation initiation factor 4 gamma 1 (EIF4G1), which was up-regulated 14 days post infection. This gene is part of the cellular translation machinery, involved in recruiting mRNA to the ribosome. Interestingly, this gene is directly targeted by the Influenza virus NS1 protein to promote viral protein translation [49], and if this interaction is compromised then viral replication is impaired [50]. EIF4G1 also interacts with the Influenza polymerase PB2 to enable cap-independent translation [51], and blocking this interaction inhibits Influenza replication [52]. Therefore, this gene is a good candidate for further investigation within the QTL for resistance to ISAV in chromosome 13. Another genomic region in chromosome 18, although not significant, contained SNP windows which explained the most genetic variance in our study (almost 5%). The closest gene to this putative QTL showing expression differences is the probable E3 ubiquitin-protein ligase HERC4 (HERC4), up-regulated both at 7 and 14 days post infection. No interactions between this gene and viral infections have been described, however viruses, including Influenza, frequently target the host ubiquitin machinery [53], and members of the same family HERC5 and HERC6 have antiviral properties in mammals. These genes attach interferon-stimulated gene 15 (ISG15) to target proteins in the presence of interferons [54], and HERC5 in fact targets the critical Influenza virulence factor NS1 protein to inhibit its replication [55]. While these genes are interesting candidates, we found no evidence of differences in their expression between ISAV resistant and susceptible fish, which might not be surprising considering the polygenic nature of ISAV resistance, the relatively small effect of these QTL and the small sample size of our resistant vs susceptible transcriptomic comparison. Additionally, these genes could show tissue-specific responses, or not depend on expression changes to have an impact on the course of the disease. Nonetheless, these genes represent potential targets for further downstream studies to try to understand the genetic factors underlying these putative QTL and more generally resistance to ISAV.

### Putative virus-induced immunomodulation of transcriptional response to ISAV in salmon

A relatively large transcriptomic response to ISAV infection was observed in the heart of Atlantic salmon at both 7 and 14 dpi. This response was characterised by a down-regulation of the complement and coagulation cascades; surprising considering the frequently observed symptoms of the disease (erythrophagia and haemorrhages [14];). In addition, a lower number of genes showed differential expression at 14 dpi than at 7 dpi, but the number of differentially expressed genes involved in the complement and coagulation cascades increased, suggesting potentially progressive immunomodulation by ISAV. Orthomyxoviruses are capable of subverting the host complement system through different mechanisms [56]. A general up-regulation of various metabolic pathways was also observed both at 7 and 14 dpi. Widespread metabolism dysregulation is commonly observed in diseased fish [57–59], although generally down-regulated and ascribed to a physiological response of the host to infection to adjust cellular homeostasis or to reduced appetite. Nonetheless, pathogens also reprogram the cellular metabolism of infected cells to favour their replication [60], and therefore the observed dysregulation could be a consequence of the host-virus interaction. In fact, the infection strategy of influenza virus includes mechanisms to alter host transcription and translation [61].

The observed interferon response was weaker than expected, with a few interferon-related genes showing lower expression levels at 7 days than in controls, and mild up-regulation of a few interferon genes at 14 dpi. For instance Mx1, which has been shown to confer resistance to ISAV in chinook salmon cells, was up-regulated at 14 dpi [62]. In general, the gene expression patterns suggest that the innate immune response was relatively mild at 7 dpi and 14 dpi. This differs from previous studies on ISAV-infected salmon [33, 34, 36, 37]. There are two possible, non-exclusive explanations for this result. First, tissue-specific regulation seems to play an important role during ISAV infection [37], and previous research has been focused in immune-tissues, which are more likely to exhibit dysregulation of genes associated with immunity. Secondly, a previous study on the response of Atlantic salmon heart to ISAV only reported significant differences in the expression of immune genes at 21 dpi, albeit some genes started to show an upward trend at 13 days [35]. Therefore, the relatively early timepoints studied here, in comparison to other studies, might affect the strength of the innate immune response observed. Our selected timepoints reflect the main goal of our study, which was to capture potential early events that might influence the outcome of the infection. A strong innate immune response may be expected in the first week following infection, but with the cohabitation challenge model used, it is difficult to predict the precise time at which individual fish become infected. Nonetheless, a clear transcriptomic response was observed, which considering the stronger response to ISAV at 7 dpi than at 14 dpi might suggest a mechanism of immunoevasion of the virus. This immunoevasion might not be effective later in the infection when viral levels are high, which is consistent with the reported positive correlation between virus abundance and the magnitude of the immune response [34, 37, 63], and is consistent with the up-regulation of several interferon genes at 14 dpi.

The down-regulation of genes involved in the interferon response (i.e. irf1, irf4 or irf8) in infected samples at 7 dpi (and lack of up-regulation of other interferon genes at this time point) suggests that ISAV modulates this process as part of its infection and replication strategy. Previous studies have demonstrated that segments 7 and 8 of the ISAV genome produce proteins with antagonist IFN properties, binding directly to IRFs [64] and down-regulating type I IFN transcription activity [65]. Segment 7 specifically inhibits the transcription of mx [66], which as mentioned above has been shown to confer resistance to ISAV in chinook salmon cells [62]. This active suppression of the interferon system is consistent with previous studies in Influenza in human and chicken [67, 68]. Additionally, SOCs and NLRc3 genes, which limit the inflammatory response [69, 70], are up-regulated in infected samples, and cytokine induction is not clearly observed, supporting the theory of the negative regulation of the innate immune response during ISAV infection. It should be noted, however, that the results of the transcriptome profiling in the current study pertain to heart tissue only, which suggests caution is required before making general interpretations. Other tissues were sampled from the same fish, and this biobank of samples could form the basis for future sequencing to give a more holistic overview of host response to ISAV, and the differences between resistant and susceptible fish.

### Transcriptomic signatures of resistance to ISAV in Atlantic salmon

Although the overall antiviral response was less striking than expected, certain interferon-related genes are up-regulated in response to ISAV (Mx1, Mx3, Irf7). Importantly, both gvinp1 and TRIM25, interferon stimulated genes (ISG), were found to have higher average expression in resistant than in susceptible samples, although the up-regulation of TRIM25 must be considered with caution since there were only two resistant fish with a very high expression of this gene. Gvinp1 is a protein directly induced by the interferon pathway, but its function is not fully understood [71, 72]. On the other hand, the function of the E3 ubiquitin / ISGA15 ligase TRIM25 is well-characterised in mammals, where TRIM25 is responsible for the ubiquitination of RIG-I, leading to the activation of the downstream pathway and increased interferon production [73], crucial for antiviral innate immunity. Interestingly, Influenza A virus non-structural protein 1 (NS1) specifically inhibits TRIM25-mediated ubiquitination of RIG-I [74]. TRIM25 can also inhibit viral RNA synthesis through direct binding to the viral RNA polymerase complex (independent of its ubiquitin ligase activity), an activity that can also be inhibited by the viral NS1 [75]. In summary, TRIM25 plays a vital role in the host response to Influenza infection in mammals, and is actively modulated by Orthomixoviruses. However, it is unclear whether this function is conserved in teleost; although fish TRIM genes show features suggesting a role in innate immunity, they show important clade-specific diversifications [76]. Nonetheless, many TRIM genes are induced upon viral infection, and some have been shown to trigger antiviral activity in vitro [77]. In common carp, a gene also annotated as TRIM25 was identified as a promising candidate for Koi herpesvirus resistance [78], which suggests its relevance in antiviral responses is well conserved.

While expression differences between resistant and susceptible fish are based on a small number of samples, and none of these genes co-localise with any of the putative QTL regions identified in this study, they represent a first layer of information towards understanding ISAV resistance. In particular, the functional relevance of TRIM25 in response to Influenza, another Orthomixovirus, suggests that this gene is a good target for future functional studies to understand and increase resistance of Atlantic salmon to ISAV.

## Conclusion

Resistance to ISAV is moderately heritable and shows a polygenic architecture amenable to genome-assisted selection schemes, albeit a significant QTL was discovered in chromosome Ssa13 explaining around 3% of the genetic variance and could be prioritised in selection schemes prior validation in follow-up studies. The heart transcriptomes of selected genetically resistant and susceptible samples revealed a complex response, which suggests a host-pathogen crosstalk regulating the innate immune response and more specifically the interferon pathway. In line with its polygenic architecture, the transcriptomic signatures of resistance are diverse in nature; nonetheless, TRIM25 could be a promising candidate for further functional studies on resistance to ISAV. Genome editing experiments should inform the role of this gene in the progression of the disease, and may help obtain salmon stocks with increased resistance to ISAV in the future, leading to increased stability, food security and fish welfare.

## Methods

### Disease challenge and sampling

The population used for the ISAV challenge experiment comprised 2833 parr fish (mean 37.5 ± 9.2 g) from 194 nuclear families originating from Benchmark Genetics breeding programme. The challenge experiment and sampling were conducted in the facilities of VESO Vikan (Norway). All fish were PIT-tagged and transferred to one 4 m^3^ tank where they were acclimated for 3 weeks in fresh water at the following approximate conditions: temperature 12 °C, stocking density 40 kg / m^3^, flow 5–6 mg O_2_ / L and photoperiod regime L:D = 24:0. Post acclimation, 300 carrier fish (Atlantic salmon from the same population) used for the cohabitation challenge were intraperitoneally injected with 0.1 mL of ISAV (Glaesvær, 080411, grown in ASK-cells, 2 passage, estimated titre 10^6^ PFU / mL [79]) and introduced to the challenge tank with the naive fish. Fish were fed using an automatic feeder during the challenge, with feeding percentage updated weekly. Fish and tanks were monitored on a daily basis, removing mortalities and moribund fish (registered as dead), and recording environmental parameters. Mortalities were registered and sampled daily, and the trial was terminated when the mortality level dropped to baseline levels (i.e. near zero). The expected clinical signs of ISA infection were observed, and a small number of mortalities (*n* = 1–3) were sampled daily and tested for ISAV using quantitative RT-PCR (qRT-PCR) to confirm the virus was the cause of death. To achieve this, a small sample of heart tissue was stored in RNA-later at − 20 °C until processing. Quantitative estimates of virus load were obtained by qRT-PCR analyses which were performed by the Fish Vet Group Norway (http://fishvetgroup.no/en/). The majority of samples tested positive and had significant ISAV titre from approximately 10 days post-challenge. Adipose fin tissue samples from all fish were collected and stored in ethanol for DNA extraction and genotyping. In addition, 30 of the challenged fish were terminated at each of three time points (pre-infection, 7 dpi and 14 dpi) for sampling of tissues for transcriptomic analyses. In addition to fin clips, the hearts of a subset of animals were collected into TRI Reagent (Sigma, UK) and stored at − 20 °C until RNA extraction.

### Genetic parameter estimation

Resistance to ISAV was measured as binary survival (BS), mortalities were recorded as 0 and survivors as 1. All challenged fish were used to estimate the genetic parameters for resistance to ISAV, using a probit link function and the ASREML software v4.1 [80]. The univariate animal model used was:

$$ \mathrm{y}=\boldsymbol{X}\mathrm{b}+\boldsymbol{Z}\mathrm{u}+\mathrm{e} $$

Where ***y*** is the vector of phenotypic records; ***b*** is the vector of fixed effects, which includes sex as fixed effect and the first two principal components of the variance-standardized relationship matrix and body weight at PIT-tagging as covariates; ***u*** is the vector of random animal genetic effects which assumes the following normal distribution ~ $\mathrm{N}\left(0,\boldsymbol{A}{\upsigma}_{\mathrm{a}}^2\right)$, where ***A*** is the additive relationship matrix and ${\upsigma}_{\mathrm{a}}^2$ is the additive genetic variance; ***e*** is the vector of residual effects with a normal distribution assumed as ~ $\mathrm{N}\left(0,\boldsymbol{I}{\upsigma}_{\mathrm{e}}^2\right)$, where ***I*** is the incidence matrix and ${\upsigma}_{\mathrm{e}}^2$ is the residual variance; and ***X*** and ***Z*** are design matrices for fixed and random effects, respectively. Heritability was estimated as:

$$ {h}^2=\frac{\sigma_a^2}{\sigma_a^2+{\sigma}_e^2} $$

Where ${\sigma}_a^2$ is the additive genetic variance and ${\sigma}_e^2$ is the estimated residual variance, which was set as 1 (Gilmour et al. 2009).

### Genotyping and GWAS

A total of 1367 samples were successfully genotyped for a 55 K Affymetrix Axiom SNP array used routinely by Benchmark Genetics in their commercial breeding programme. DNA extraction from fin clips and SNP array genotyping was performed by IdentiGEN (Dublin, Ireland). Quality control (QC) was performed using PLINK software v1.90 [81]. SNPs with minor allele frequency (MAF) lower than 0.05 or significantly deviating from Hardy–Weinberg Equilibrium (HWE) (*p* < 1e-6) were removed for further analyses; SNPs and individuals with a call rate lower than 99% were also excluded.

A weighted single-step GBLUP approach (wssGBLUP) was used to estimate genomic heritability and to identify the genomic regions associated with ISAV resistance. This approach uses all animals with phenotypic data, connecting genotyped and not genotyped fish through the pedigree [82]. Pedigree and genotypic information are combined to create an ***H*** matrix [83]. Thus, the inverse of this ***H*** matrix is:

$$ {\boldsymbol{H}}^{-1}={\boldsymbol{A}}^{-1}+\left[\begin{array}{cc}0& 0\\ {}0& {\boldsymbol{G}}^{-1}-{\boldsymbol{A}}_{22}^{-1}\end{array}\right] $$

Where ***A***^−1^ is the inverse of the pedigree-based relationship matrix, ${\boldsymbol{A}}_{22}^{-1}$ represents the inverse of the ***A*** matrix, but only considering the genotyped fish, and ***G***^−1^ is the inverse of the genomic relationship matrix. The statistical model for the genetic parameter estimation and for the genome-wide association study is identical as the one mentioned above, but replacing the ***A*** matrix by the **H** matrix. Genomic heritability using the **H** matrix was estimated as described above. The variance obtained on the first iteration of wssGBLUP for each SNP (single-step GWAS) was used as the weight in the analyses. The variances were estimated based on the allele frequency and marker effect [84]. A threshold model was fitted for BS using the THRGIBSS1F90 function of BLUPF90 [85], and a total of 200,000 Markov Chain Monte Carlo (MCMC) iterations were fitted. From these, 20,000 were burned-in, and 1 from every 50 of the remaining 180,000 samples were saved.

Additionally, a mixed linear model, using the leaving-one-chromosome-out (LOCO) approach was fitted to identify SNPs associated with resistance to ISAV, through the GCTA v.1.92.2. software [86]. The fitted model was identical as the one described for the wssGBLUP approach, although a G matrix was used. For a SNP to be significantly associated at genome-wide level with resistance to ISAV, it must surpass the Bonferroni-corrected significance threshold (α/n), where a and n represent the significance level (0.05) and the number of SNP that surpassed the QC, respectively.

Finally, the *p*-values for each SNP and the proportion of the genetic variance explained by 20 adjacent SNP window were plotted with R/CMplot.

The wssGBLUP approach was used for the estimation of the genetic parameters and the genome-wide association study was also used to predict the genomic estimated breeding values (gEBVs) for resistance to ISAV, estimating the genetic resistance and susceptibility of the fish sampled for transcriptomic experiments.

### RNA extraction and RNA sequencing

For each timepoint (control, 7 dpi and 14 dpi) 4 fish with high breeding values for resistance and 4 fish with low breeding values for resistance, representing 8 different families, were selected according to their estimated genomic breeding value for ISAV resistance. Heart RNA was extracted from preserved tissue samples (*n* = 24; 8 x controls, 8 × 7 dpi, 8 × 14 dpi) in TRI Reagent (Sigma, UK) and RNA extracted following the manufacturer’s instructions. The RNA pellet was eluted in 15 μL of nuclease-free water and quantified on a Nanodrop 1000 spectrophotometer (NanoDrop Technologies) prior to DNAse treatment with QuantiTect® Reverse Transcription kit (Qiagen). The quality of the RNA was examined by electrophoresis on a 1% agarose gel (Sigma Aldrich), prepared in Tris-Acetate-EDTA (TAE) buffer, stained with 1% SYBR Safe (Sigma Aldrich) and run at 80 V for 30 min. Sample concentration was measured with Invitrogen Qubit 3.0 Fluorometer using the Qubit RNA HS Assay Kit (ThermoFisher Scientific). PolyA RNA-Seq libraries were prepared using Illumina’s TruSeq RNA Library Prep Kit v2 by Oxford Genomic Centre, and sequenced on an Illumina Novaseq6000 as 150 bp paired-end reads yielding an average of 51 M reads per sample (minimum 38 M).

### RNA-Seq analyses

Raw reads were quality trimmed using Trimgalore v0.6.3. Briefly, adapter sequences were removed, low quality bases were filtered (Phred score < 20) and reads with less than 20 bp were discarded. Trimmed reads were then pseudoaligned against the Atlantic salmon reference transcriptome (ICSASG_v2 Annotation Release 100) [87] using kallisto v0.44.0 [88]. Transcript level expression was imported into R v3.6 [89] and summarised to the gene level using the R/tximport v1.10.1 [90]. Differential expression analysis was performed using R/Deseq2 v1.22.2 [91], and genes with False Discovery Rate adjusted *p*-values < 0.05 were considered to be differentially expressed. Kyoto Encyclopedia of Genes and Genomes (KEGG) enrichment analyses were carried out using KOBAS v3.0.3 [92]. Briefly, salmon genes were annotated against the KEGG protein database [93] to determine KEGG Orthology (KO). KEGG enrichment for differentially expressed gene lists was tested by comparison to the whole set of expressed genes (average of > 10 normalised reads) in the corresponding tissue using Fisher’s Exact Test. KEGG pathways with ≥5 DE genes assigned and showing a Benjamini-Hochberg FDR corrected *p*-value < 0.05 were considered enriched.

### Data availability

RNA sequencing raw reads have been deposited in the NCBI’s Short Read Archive (SRS) repository with accession number PRJNA647285. The DESeq2 normalised gene expression matrix is available as supplementary file 1.

## Supplementary Information

**Additional file 1.**

**Additional file 2.**

**Additional file 3.**

**Additional file 4.**

**Additional file 5.**

**Additional file 6.**

**Additional file 7.**

## Data Availability

The datasets generated and analysed during the current study are available in the NCBI’s Short Read Archive (SRS) repository with accession number PRJNA647285.
