# Supplementary figures and images for: Exploring genetic resistance to infectious salmon anaemia virus in Atlantic salmon by genome-wide association and RNA sequencing
Source: BMC Genomics. 2021 May 13;22:345. doi: 10.1186/s12864-021-07671-6 (PMC8117317; doi:10.1186/s12864-021-07671-6)

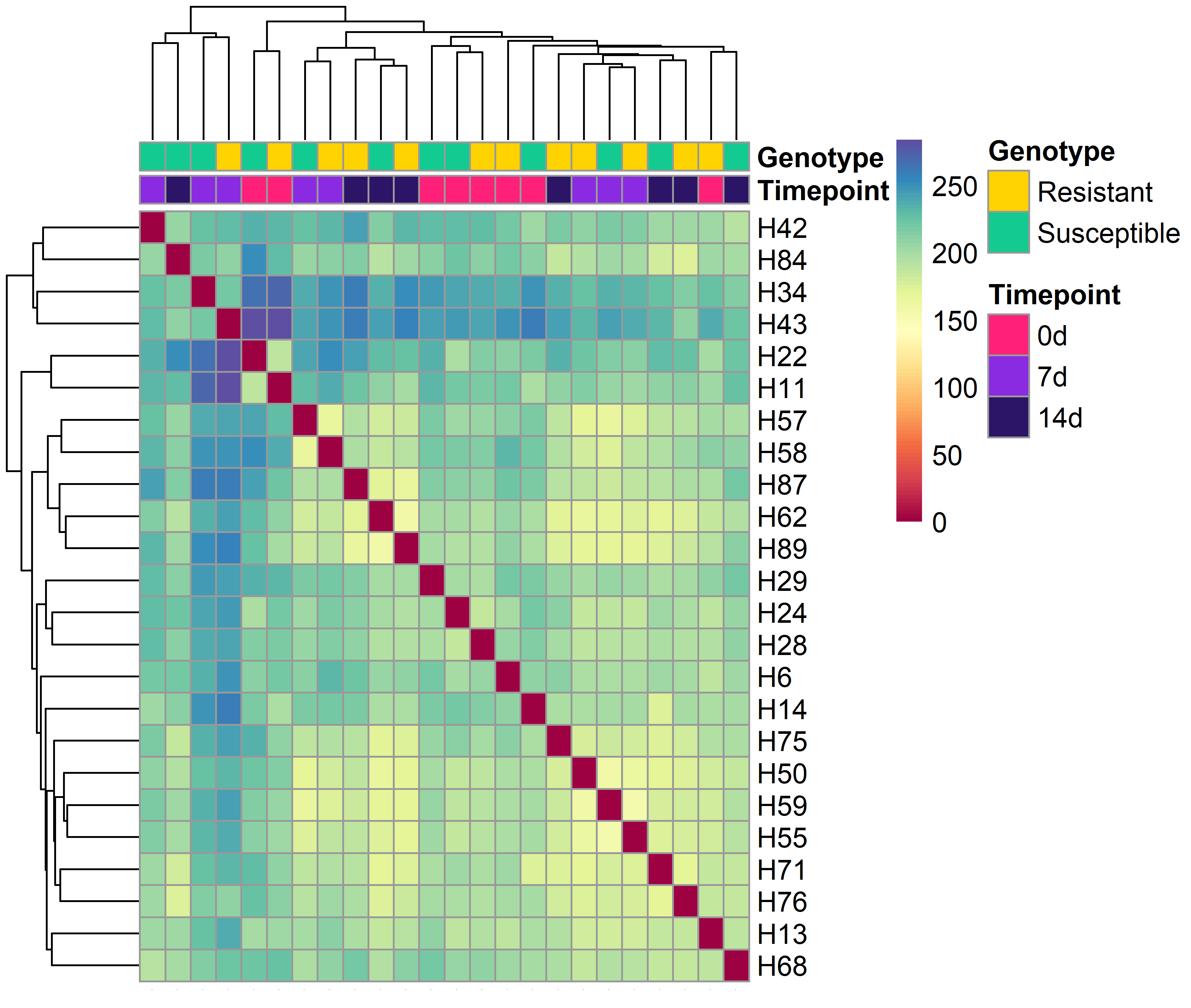

Supplement: Supplementary file 1 — Additional file 1. [file 12864_2021_7671_MOESM1_ESM.png]
